# Canalization and developmental stability of the yellow-necked mouse (*Apodemus flavicollis*) mandible and cranium related to age and nematode parasitism

**DOI:** 10.1186/s12983-021-00439-4

**Published:** 2021-10-24

**Authors:** Vida Jojić, Borislav Čabrilo, Olivera Bjelić-Čabrilo, Vladimir M. Jovanović, Ivana Budinski, Mladen Vujošević, Jelena Blagojević

**Affiliations:** 1 Department of Genetic Research, Institute for Biological Research “Siniša Stanković” - National Institute of Republic of Serbia, University of Belgrade, Belgrade, Serbia; 2 Department of Biology and Ecology, Faculty of Sciences, University of Novi Sad, Novi Sad, Serbia; 3 Bioinformatics Solution Center, Freie Universität Berlin, Berlin, Germany; 4 Human Biology and Primate Evolution, Freie Universität Berlin, Berlin, Germany

**Keywords:** Covariation, Developmental homeostasis, Developmental instability, Fluctuating asymmetry, 2D Geometric morphometrics, Intestinal helminths, Mammals, Skull, Postnatal ontogeny, Rodents

## Abstract

**Background:**

Mammalian mandible and cranium are well-established model systems for studying canalization and developmental stability (DS) as two elements of developmental homeostasis. Nematode infections are usually acquired in early life and increase in intensity with age, while canalization and DS of rodent skulls could vary through late postnatal ontogeny. We aimed to estimate magnitudes and describe patterns of mandibular and cranial canalization and DS related to age and parasite intensity (diversity) in adult yellow-necked mice (*Apodemus flavicollis*).

**Results:**

We found the absence of age-related changes in the levels of canalization for mandibular and cranial size and DS for mandibular size. However, individual measures of mandibular and cranial shape variance increased, while individual measures of mandibular shape fluctuating asymmetry (FA) decreased with age. We detected mandibular and cranial shape changes during postnatal ontogeny, but revealed no age-related dynamics of their covariance structure among and within individuals. Categories regarding parasitism differed in the level of canalization for cranial size and the level of DS for cranial shape. We observed differences in age-related dynamics of the level of canalization between non-parasitized and parasitized animals, as well as between yellow-necked mice parasitized by different number of nematode species. Likewise, individual measures of mandibular and cranial shape FA decreased with age for the mandible in the less parasitized category and increased for the cranium in the most parasitized category.

**Conclusions:**

Our age-related results partly agree with previous findings. However, no rodent study so far has explored age-related changes in the magnitude of FA for mandibular size or mandibular and cranial FA covariance structure. This is the first study dealing with the nematode parasitism-related canalization and DS in rodents. We showed that nematode parasitism does not affect mandibular and cranial shape variation and covariance structure among and within individuals. However, parasite intensity (diversity) is related to ontogenetic dynamics of the levels of canalization and DS. Overall, additional studies on animals from natural populations are required before drawing some general conclusions.

**Supplementary Information:**

The online version contains supplementary material available at 10.1186/s12983-021-00439-4.

## Background

Developmentally and functionally complex morphological structures, such as mammalian mandible and cranium, are well-established model systems for studying canalization, developmental stability (DS), and morphological integration, recognized by Hallgrímsson et al. [1] as three main components of phenotypic variability. Canalization and DS also represent two elements of developmental homeostasis or homeorhesis, the mechanism responsible for ensuring phenotypic constancy in organisms despite the great variability of genetic and environmental features [2–4]. According to early explanations of canalization and DS, the former is considered as the buffering of developmental processes against environmental and mutational perturbations, and the latter as the ability of developmental processes to buffer random developmental noise that arises within the developmental processes themselves [5, 6]. Similarity between canalization and DS lies in the fact that both limit phenotypic variation. The key distinction between them is in the origin of potential factors contributing to phenotypic variation in such a way that environmental perturbations arise outside of an individual, while developmental noise, as stochastic fluctuations in developmental processes, arises within an individual [1, 7–9]. Although developmental noise is not caused by environmental or genetic variation, environmental and genetic factors can affect developmental processes that mediate its expression and thus influence DS [9, 10].

Symmetric traits in different individuals develop under different genetic and environmental conditions, whereas in the same individuals they share the same genome and experience the same environmental conditions [8, 9]. Therefore, the deviations from perfect symmetry are caused by developmental disruptions arising within an individual. Consequently, the differences in the amount of among-individual variation (Ind) indicate differences in ability to canalize development against genetic and environmental stresses, while the differences in the amount of within-individual variation or fluctuating asymmetry (FA), i.e. random differences between the two sides in bilaterally symmetric traits [11, 12], indicate differences in ability to buffer development against random developmental noise. Thus, the amount of Ind is used to measure canalization and the amount of FA serves as the measure of DS. Lower level of Ind and FA signify a higher level of canalization and DS, respectively. Because ancestral states of canalization and DS for a structure are unknown, they can only be estimated by comparison with some reference state [8, 13].

The factors that may disturb developmental homeostasis can be of environmental or genetic origin [14]. Traditional and geometric morphometric methods have been used frequently in a wide range of organisms, from plants to humans, for examining whether various environmental and genetic factors disrupt the level of canalization and DS [1, 4, 9, 12, 14–20]. In the 1990s geometric morphometric methods offered new tools for exploring patterns of morphological variation, i.e. covariance structures, both among and within individuals, in relation to potential environmental and genetic stressful factors, as well as for inspecting relationships between canalization and DS [1, 9, 20–22]. Ontogenetic dynamics of magnitude and pattern of canalization/DS [1, 23–26], as well as their associations with fitness [1, 7, 9, 14, 20] have also been examined.

Within rodents, canalization and DS of skull traits were explored using laboratory-bred mice (*Mus musculus domesticus*; *Calomys expulsus*) and rats (*Sigmodon fulviventer*; *Rattus norvegicus albinus*), as well as rodents from wild populations. Laboratory-based studies delivered important knowledge regarding relations between magnitude/pattern of variation among and within individuals and environmental [27–30] or genetic [18, 31–34] factors, relationships between patterns of Ind and FA as evidence of whether the same developmental mechanisms generate canalization and DS [21], ontogenetic changes in magnitude of variation and covariance structure among individuals [23–25, 35–37], and genetic architecture of FA [38]. Considering studies that involved specimens from wild rodent populations, the majority of them investigated associations between FA levels of meristic [39, 40] and metric [41–47] mandibular and cranial characteristics with environmental and genetic perturbations, while few of them compared patterns between Ind and FA to infer whether developmental mechanisms behind canalization and DS are closely related [45, 48, 49]. Moreover, examinations of parasite (viral) infections effects on host (rodents from wild populations) morphology found that viruses can disrupt host development affecting mean cranial shape and canalization [50, 51].

Although numerous studies have reported a relationship between DS and parasite infections, reflected in increased levels of FA in host individuals [14, 17, 52], inside vertebrate groups this association was studied mostly in some fish and avian species, whereas within mammals it has so far been examined only in humans and reindeer (*Rangifer tarandus*) [17, 52]. According to Møller [52], there are at least three reasons responsible for this relationship. First, individuals with elevated levels of FA may be more susceptible to parasitism; host FA would reflect an inefficiency of the immune system to resist infection [52–54]. Second, individuals with higher levels of FA may more often be exposed to parasites; if competitive ability depends on body condition, developmentally unstable individuals will more often be restricted to poor environments with elevated risk of encountering parasites [14, 52, 54, 55]. Third, parasites may disrupt host development and be the direct cause of instability [52]; host FA should be directly related to parasite virulence, parasite load, or both [54, 56–58].

The yellow-necked mouse (*Apodemus flavicollis* Melchior, 1834), common in the Western Palearctic region, is abundantly distributed throughout the territory of Serbia. The nematode fauna of *A. flavicollis* is well documented in several European countries [59–62], as well as in Serbia [63–66]. Additionally, this mouse species is characterized by the presence of the oldest known chromosome polymorphism [67], i.e. supernumerary or B chromosomes (Bs) [68, 69]. Therefore, wild-caught *A. flavicollis* has been used as a model organism for various surveys of Bs in mammals [70, 71], while its mandible and cranium have been used as model systems not only for examining phenotypic effects of Bs [72–74], including those on canalization, DS and morphological integration [40, 45, 75], but also for exploring morphological modularity and comparison of traditional and geometric morphometric procedures for analyzing it [76]. Earlier investigations in *A. flavicollis* found that Bs do not disturb developmental homeostasis in their carriers [40, 45], but they play a significant role in structuring cranial variation [45]. In addition, after analyzing the possible influence of Bs on genes involved in immune response and helminth burden in *A. flavicollis*, Adnađević et al. [77] concluded that animals with and without Bs have the same endpoint immune response to parasites yet achieved through different pathways.

Our initial aim in the present study was to inspect canalization and DS of the mandible and cranium in relation to intestinal nematode parasitism in adult specimens of *A. flavicollis* from the territory of Serbia. Moreover, nematode infections are usually acquired in early life and increase in intensity with age [77, 78], while canalization and DS of rodent skulls could vary through late postnatal ontogeny [23–25, 35–37]. Consequently, we aimed to examine canalization and DS of the mandible and cranium in relation to age of *A. flavicollis* adults. Thus, both magnitude (for size and shape) and pattern (for shape) of variation among and within individuals were explored, and compared, separately across age categories and categories regarding parasitism.

With regards to the age-related dynamics of canalization and DS, and in line with previous findings in laboratory-reared rodents, we tested the following hypotheses: (1) Variance among individuals (for mandibular and cranial size and shape) will decrease with age, i.e. the highest levels of canalization for mandibular and cranial size and shape will be in the oldest yellow-necked mice; (2) There will be no change in the magnitude of shape variation within individuals through late ontogeny, i.e. we assume the absence of age-related dynamics of the levels of DS for mandibular and cranial shape; (3) Mandibular and cranial shape will differ across ontogenetic stages, as well as mandibular and cranial covariance structure among individuals. To the best of our knowledge, no rodent study has explored either changes in the magnitude of mandibular size variation within individuals through late ontogeny, or ontogenetic dynamics of mandibular and cranial FA covariance structure, so we could not make any predictions.

Regarding the relationship of nematode parasitism with canalization and DS, with no assumption of any of the three reasons proposed by Møller [52], we tested the following hypotheses: (1) Infections by intestinal nematodes may be associated with the levels of canalization and DS in *A. flavicollis*. Thus, we expect that parasitized animals possess lower levels of canalization (for mandibular and cranial size and shape) and DS (for mandibular size and mandibular and cranial shape) compared to non-parasitized yellow-necked mice; and (2) Infections by intestinal nematodes may be linked with the shape variation among individuals and the patterns of variation among and within individuals of *A. flavicollis*. Accordingly, we expect differences in the mandibular and cranial shape variation among individuals, as well as differences in mandibular and cranial patterns of canalization and DS, between different categories of parasitized animals.

## Methods

### Data collection and sample composition

**Fig. 1:**
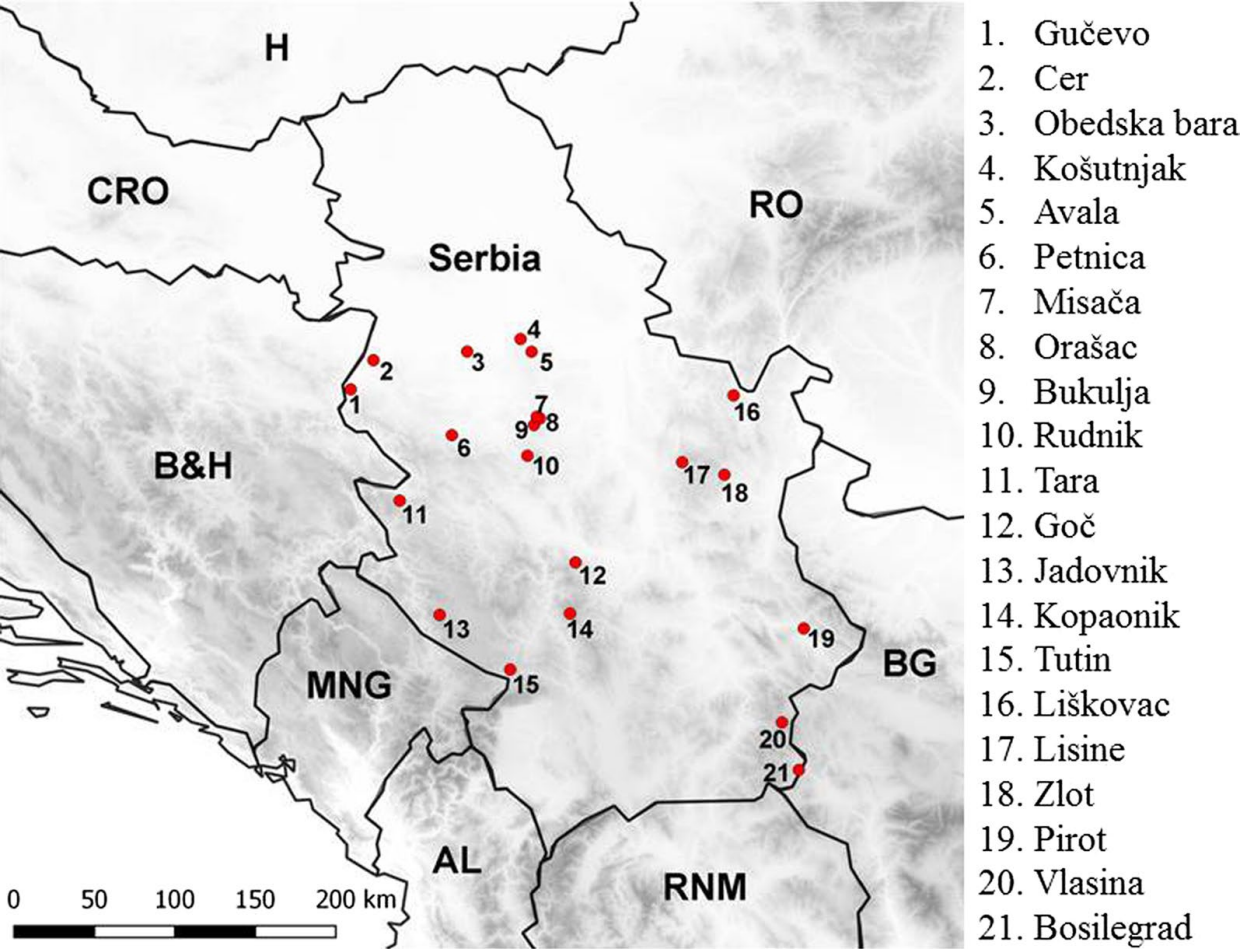
Geographic distribution of the yellow-necked field mouse (*Apodemus flavicollis*) localities from Serbia

Yellow-necked mice (*Apodemus flavicollis*) were collected between 2011 and 2015 from 21 localities in Serbia (Fig. 1). Mice were trapped using Longworth traps baited with sardines and wheat. During dissection, each animal was sexed by gonad inspection. ISSR-PCR method was used to distinguish *A. flavicollis* from *A. sylvaticus* [79]. Chromosomes were prepared directly from bone marrow according to the standard procedure [80]. The presence of B chromosomes (Bs) was determined by analyzing 30 metaphase figures per specimen using counting tool in software Analyst [81]. All animals characterized by more than 48 chromosomes (standard chromosome set) were considered to have Bs. The age of each individual was estimated based on the weight of dry eye lens [82]. The intestinal tract was dissected from each animal and nematode parasites (*Aonchotheca annulosa*, *Aspiculuris tetraptera*, *Eucoleus* sp., *Heligmosomoides polygyrus*, *Mastophorus muris*, *Rictularia proni*, *Syphacia frederici*, *S. stroma*, and *Trichuris muris*) were identified using keys by Ryzhikov et al. [83] and Genov [60]. The yellow-necked mouse sample consisted of uninfected and infected individuals, with the latter hosting one to five different species of intestinal nematodes [64]. Mandibles and crania were cleaned by exposure to dermestid beetles.

**Fig. 2:**
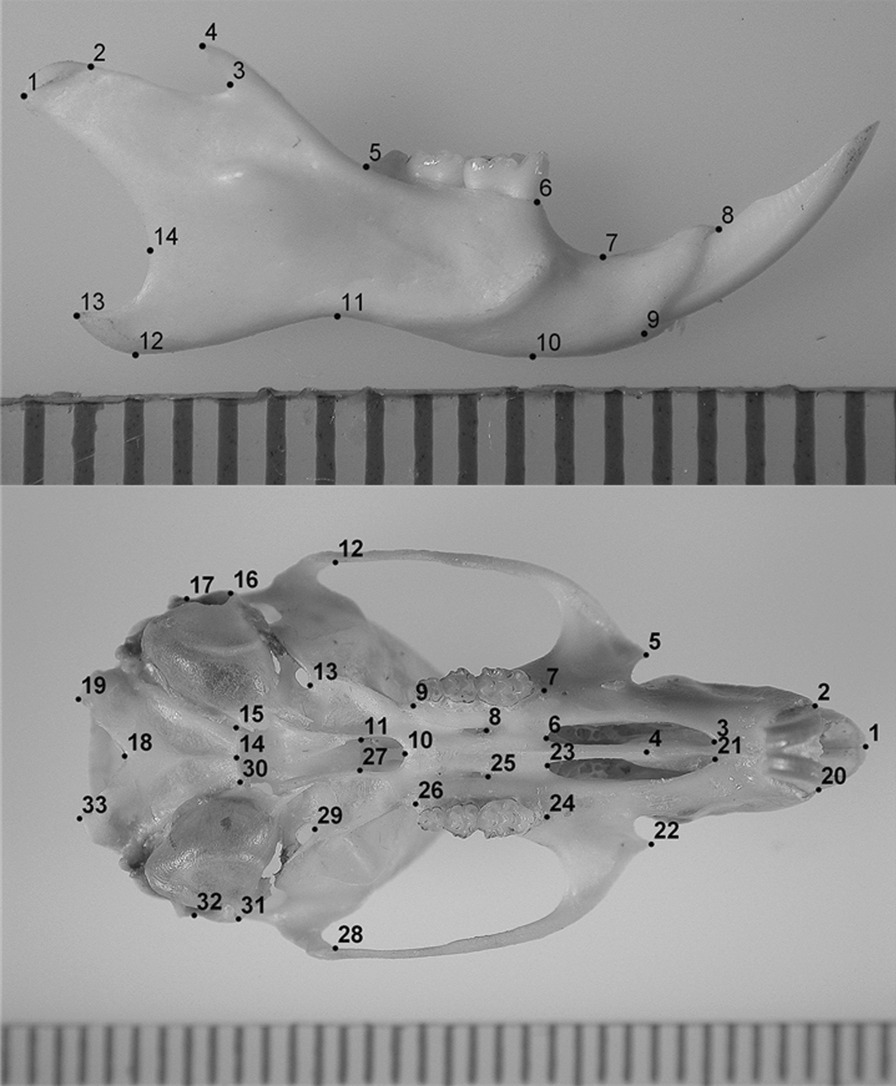
Landmarks recorded on the labial view of the mandible and the ventral surface of the cranium of the yellow-necked field mouse (*Apodemus flavicollis*). See Additional file 1: Table S1, for landmark definitions

We studied a total of 275 mandibles and 320 crania of adult animals. Images (2272 × 1520 pixels resolution) of left and right mandibles in the labial view and images (2272 × 1520 pixels resolution) of crania in the ventral view, supported by modeling clay with the palate positioned parallel to the photographic plane, were taken with a NIKON Coolpix 4500 digital camera by the same person (VJ). Mandibles are more fragile than crania and during cleaning and imaging some of them were damaged, particularly in the region of the coronoid process. As studies of mandibular fluctuating asymmetry (FA) require both intact mandibles (left and right), their sample size was smaller than sample size of crania. Using TpsDig software [84, 85], the same observer (VJ) recorded 14 and 33 (14 paired and five median) two-dimensional landmarks on the mandible and cranium, respectively (Fig. 2). Anatomical definitions of landmarks are given in Additional file 1: Table S1.

Prior to digitizing 2D landmarks on mandibles, all images of left mandibles were reflected to right side to avoid a bias when locating landmarks on images of the left and right sides of biological structures [20]. To assess measurement error due to positioning and imaging of 3D biological structures such as the cranium, we conducted Procrustes analysis of variance (ANOVA) [86, 87] for a set of 96 yellow-necked mice (29.7% of the total). Two images of each cranium were taken, and each image was digitized twice. The mean square for FA and individual variation exceeded the error component (effect of imaging) by fivefold and 64-fold, respectively. Therefore, all the subsequent analyses were based on a single image per cranium. However, as replicates are necessary for studies of FA, each image of the right and the mirror-reflected left mandible, as well as each image of the cranium, was digitized twice in two separate sessions.

In an earlier study concerning sex ratio variation of intestinal nematode infrapopulations in *A. flavicollis*, Jovanović et al. [66] distinguished three age categories of host mice, estimated from dry eye lens weight. Based on the same criterion, we also recognized the same three age categories of adult *A. flavicollis*, i.e. animals whose dry eye lens mass in mg was: ≤ 15 mg (A1 category), 15–25 mg (A2 category), and > 25 mg (A3 category) (Table 1). According to the presence and number of nematode species detected within a single individual (parasite diversity), we recognized four categories regarding parasitism: non-parasitized animals (P0 category), animals parasitized by one (P1 category), two (P2 category), and three to five (P3 category) nematode species (Table 1). Dealing with limited sample sizes of adult wild-caught specimens of *A. flavicollis* belonging to particular categories regarding parasitism and age (overall 12 categories whose sample sizes are too small for statistical tests), we were in a rather difficult situation to separate effects of parasitism from the effects of aging on canalization and DS. Consequently, all the following analyses were performed separately for age categories and categories regarding parasitism.

**Table 1:** Sample composition

		(P0, P1, P2, P3)	(m, f)	(B0, B +)
Mandible	A1	(29, 29, 16, 3)	(37, 40)	(57, 20)
	A2	(15, 41, 36, 18)	(63, 47)	(72, 38)
	A3	(2, 26, 34, 26)	(39, 49)	(56, 32)
Cranium	A1	(41, 36, 27, 3)	(52, 55)	(77, 30)
	A2	(13, 42, 45, 19)	(71, 48)	(79, 40)
	A3	(2, 28, 36, 28)	(44, 50)	(61, 33)

A1—first age category (animals with dry eye lens mass ≤ 15 mg), A2—second age category (animals with 15–25 mg of dry eye lens mass), A3—third age category (animals with dry eye lens mass > 25 mg) according to Jovanović et al. yyyy[66]; P0—non-parasitized animals, P1—animals parasitized by one nematode species, P2—animals parasitized by two nematode species, P3—animals parasitized by three to five nematode species. m—males, f—females; B0—animals with standard karyotype (without B chromosomes, Bs), B + – animals with Bs

### Preliminary analyses

Spearman’s rank correlation coefficient revealed statistically significant relationship between categories regarding parasitism and age categories in both mandible (r_s_ = 0.413; P < 0.05) and cranium (r_s_ = 0.423; P < 0.05) samples, indicating that parasite infection (parasite diversity) is higher in older animals. Additionally, we calculated body condition indices (BCI) as the residuals of linear regression of total body mass on standard length [88]. One-way analysis of variance (ANOVA) showed no difference in BCI among categories regarding parasitism (F_3, 316_ = 1.70, P = 0.1672), indicating that parasite diversity does not affect the body condition of animals.

### Size analyses

Within each category, the landmark coordinates of original and mirrored configurations of both replicates were aligned simultaneously using a generalized Procrustes analysis (GPA) [89–91] to extract centroid size (CS) and superimposed Procrustes coordinates. Procrustes ANOVA [86, 87] was performed to partition overall size variation into individual variation (effect of Individuals, Ind), directional asymmetry (effect of Side, DA), fluctuating asymmetry (effect of Individual-by-Side interaction, FA), measurement error component (effect of digitizing), and variation caused by additional main effects of sex and presence of B chromosomes (Bs). Parametric F-tests in ANOVA of CS were used to determine whether size variation among individuals, DA, FA, and additional effects of sex and presence of Bs were significant within each category. For datasets with object symmetry, i.e. cranial datasets, there are no effects of Side or Individual-by-Side interaction, nor is there asymmetric CS (size differences between sides) due to the fact that CS is computed for the entire configuration (including both sides) [87]. To check for potential mandibular size antisymmetry, asymmetric CS (size differences between left and right mandibles) was inspected for signs of bimodality using the Kolmogorov–Smirnov test of normality [12, 92].

For mandibular and cranial size, the level of canalization was assessed by among-individual size variance [18, 23, 25]. Mandibular among-individual size variance was calculated as the variance of symmetric CS (Var_SymmCS_), while cranial among-individual size variance was calculated as the variance of CS (Var_CS_). To estimate the significance of differences in size variance among categories, Levene’s test was performed on absolute size differences (SymmCS_j_—SymmCS_mean_ for the mandible; CS_j_—CS_mean_ for the cranium).

For mandibular size, the level of developmental stability (DS) was estimated by the level of size FA using FA10a index [12, 30]. To estimate the significance of differences in mandibular size FA among categories, Levene’s test was performed on asymmetric CS.

In addition, to determine the association of individual measures of mandibular and cranial size variance and mandibular size FA with age, absolute size differences and asymmetric CS were regressed on dry eye lens mass (age) within the whole sample for age categories, as well as within each category regarding parasitism.

### Shape analyses

Within each category, to eliminate the influence of allometry, all individuals were standardized to the mean CS for corresponding category using the Standard6b program [93]. Subsequently, Procrustes ANOVA [86, 87] was performed to partition overall shape variation into individual variation, directional asymmetry, fluctuating asymmetry, measurement error component, and variation caused by additional main effects of sex and presence of B chromosomes (Bs). Parametric F-tests in Procrustes ANOVA were used to determine whether shape variation among individuals, DA, FA, and additional effects of sex and presence of Bs were significant. To check for potential shape antisymmetry, we visually examined the plots of scores for the first five PCs of the vectors of individual signed asymmetries, which explained more than 50% of the asymmetric component of shape variation. If there is bimodality in the data, there should be a clustering of the data points. Additionally, each of the five PCs scores was inspected for signs of bimodality using the Kolmogorov–Smirnov test.

For mandibular and cranial shape, the level of canalization was estimated by among-individual shape variance [18, 23, 25]. Variance in shape (Var_shape_) was calculated for Procrustes distances (Pds), computed from the symmetric component of shape variation, based on the standard metric for variance. To assess the significance of the differences in shape variance among categories, Levene’s test was performed on these Pds.

For mandibular and cranial shape, the level of DS was estimated by the level of shape FA using FA10a index of Palmer & Strobeck [12]. To assess the significance of differences in shape FA among categories, Levene’s test was performed on shape FA scores (in units of Pd) calculated from the asymmetric component of shape variation [18, 23, 25, 30].

In addition, to determine the association of individual measures of mandibular and cranial shape variance and FA with age, Pds and shape FA scores were regressed on dry eye lens mass (age) within the whole sample for age categories, as well as within each category regarding parasitism.

Previously size-standardized datasets were combined in a single dataset and averaged by individual. To quantify the mean shape differences between categories in the symmetric component of shape variation (individual variation, Ind), Pds were computed. To assess their statistical significance we used a permutation test (with 10 000 permutation runs) under the null hypothesis of equal category means, followed by Bonferroni adjustment. Afterward, principal component analysis (PCA) was performed on the covariance matrix of symmetric component of shape variation and displayed as a scatterplot of the first two principal components (PC1 and PC2) [25]. TPS deformation grids [94] were used to visually detect the magnitude and direction of Ind shape changes among categories separated along PC1 axis. As presented in Table 1, within P1 and P2 categories the number of specimens of A1, A2, and A3 categories is more or less balanced, whereas P0 category mostly comprises the youngest (A1) and P3 category almost completely consists of older (A2 and A3) individuals. To decipher parasitism- and age-related changes in the symmetric component of shape variation we conducted discriminant function analysis (DFA) between P0 and P3 categories and multivariate regression of their shape variables onto dry eye lens mass.

To test whether categories differ in the pattern of symmetric (Ind) and asymmetric (FA) shape variation, we compared the respective covariance matrices by matrix correlation. The significance of matrix correlation was obtained using the matrix permutation test with 10 000 iterations against the null hypothesis of complete dissimilarity between the respective covariance matrices [95], by permuting landmarks and including the diagonal entries of the matrices.

Spearman’s rank correlation, Kolmogorov–Smirnov and Levene’s tests, as well as ANOVA and regressions, were performed using Statistica v. 5.1 [96]. All other analyses were done in MorphoJ [97].

## Results

### Age categories—Size

Parametric F-tests in ANOVAs of centroid size (CS) reveal that mandibular and cranial size variation among individuals, as well as directional asymmetry (DA) and fluctuating asymmetry (FA) for the mandible, are all highly significant in all age categories (Additional file 2: Table S2). Additional effect of sex is significant in A2 and A3 categories, whereas effect of presence of B chromosomes (Bs) is insignificant within each age category. Although significant, DA accounts for a fairly small percentage of the total mandibular size variation (0.40%, 0.26%, and 0.49% in A1, A2, and A3 category, respectively). Kolmogorov–Smirnov tests disclose that CS asymmetry is normally distributed, indicating the absence of antisymmetry in mandibular size within each age category.

Both variance of symmetric CS (Var_SymmCS_) for mandibular size and variance of CS (Var_CS_) for cranial size are the highest in A2 category, followed by A3 and A1 for the mandible and A1 and A3 for the cranium (Table 2). Levene’s tests performed on absolute size differences (SymmCS_j_—SymmCS_mean_ for the mandible and CS_j_—CS_mean_ for the cranium) reveal no significant differences in size variance among age categories (mandible: F_2, 272_ = 0.98, P = 0.3753; cranium: F_2, 317_ = 2.44, *P* = 0.0885). Besides, regressions of absolute size differences on dry eye lens mass show no age-related changes in mandibular and cranial size variance (mandible: r = 0.0366, *P* = 0.5455; cranium: r = 0.0099, *P* = 0.8600).

**Table 2:** Levels of canalization and developmental stability (DS) for the mandible and cranium

	Var_SymmCS_	Var_CS_	Var_shape_	SizeFA10a	ShapeFA10a
Mandible					
A1	2589.74	–	0.00102	6.04	0.00414
A2	3349.65	–	0.00109	5.89	0.00357
A3	2866.80	–	0.00116	8.46	0.00345
Cranium					
A1	–	7641.06	0.00037	–	0.00134
A2	–	9658.27	0.00038	–	0.00129
A3	–	7434.28	0.00043	–	0.00140

Age categories: A1—first age category, A2—second age category, A3—third age category. Var_SymmCS_—mandibular among-individual size variance; Var_CS_—cranial among-individual size variance; Var_shape_—variance in mandibular/cranial shape; SizeFA10a—level of fluctuating asymmetry (FA) for mandibular size; ShapeFA10a—level of fluctuating asymmetry (FA) for mandibular/cranial shape. FA10a = 0.798√MS_sj_-MS_m_ calculated from the values given in Additional files 2 and 3: Tables S2 and S3

For mandibular size the level of FA (SizeFA10a index) is the highest in A3 and the lowest in A2 category (Table 2). Levene’s test performed on asymmetric CS discloses no statistically significant differences in the level of mandibular size FA among age categories (F_2, 272_ = 0.91, *P* = 0.5838). Moreover, regression of asymmetric CS versus dry eye lens mass indicates the absence of the association of individual measures of size FA with age (r = −0.0332, *P* = 0.5838).

### Age categories—shape

As shown by parametric F-tests in Procrustes ANOVAs of shape (Additional file 3: Table S3), mandibular and cranial shape variation among individuals, as well as directional asymmetry (DA) and fluctuating asymmetry (FA), are all highly significant in all age categories. Although additional effect of sex is significant only for the mandible in A1 and A3 categories, it accounts for a smaller percentage of the total shape variation than individual variation and FA (A1 category: Sex = 2.10%; Individual = 76.31%; Ind × Side = 14.71%; A3 category: Sex = 1.76%; Individual = 82.77%; Ind × Side = 9.88%). Additional effect of presence of B chromosomes (Bs) is significant only for the mandible in A1 category where it accounts for smaller percentage of the total shape variation than individual variation and FA (Bs = 1.64%; Individual = 76.31%; Ind × Side = 14.71%). Although significant, DA accounts for a fairly small percentage of the total mandibular and cranial shape variation (mandible: 1.06%, 1.41%, and 1.47% in A1, A2, and A3 category, respectively; cranium: 1.90%, 1.62%, and 1.83% in A1, A2, and A3 category, respectively). In all age categories the visual examinations of the scatter plots for the first five PCs of the vectors of individual signed asymmetries reveal no evidence for clustering of the data points. Additionally, Kolmogorov–Smirnov tests indicate that distributions of each of the five PCs scores don’t show a significant departure from normality. Thus, these asymmetries can be assigned to FAs.

For both mandible and cranium, among-individual shape variance (Var_shape_) is the lowest in A1 and the highest in A3 category (Table 2). Levene’s tests performed on Procrustes distances (Pds) reveal no significant differences in shape variance among age categories (mandible: F_2, 272_ = 0.39, *P* = 0.6769; cranium: F_2, 317_ = 1.81, *P* = 0.1654). However, regressions of Pds on dry eye lens mass are statistically significant (mandible: r = 0.1241, *P* = 0.0397; cranium: r = 0.1504, *P* = 0.0070) indicating their increase with age.

For mandibular shape the level of FA (ShapeFA10a index) is the highest in A1 and the lowest in A3 category, whereas for cranial shape the level of FA (ShapeFA10a index) is the highest in A3 and the lowest in A2 category (Table 2). Levene’s tests performed on shape FA scores unveil no significant differences in the level of mandibular and cranial shape FA among age categories (mandible: F_2, 272_ = 2.13, *P* = 0.1211; cranium: F_2, 317_ = 0.66, *P* = 0.5162). However, regression of mandibular Procrustes FA scores versus age is statistically significant (r = − 0.1667, *P* = 0.0056) indicating their decline with age, while regression of cranial Procrustes FA scores versus age is not statistically significant (r = 0.0836, *P* = 0.1359).

**Fig. 3:**
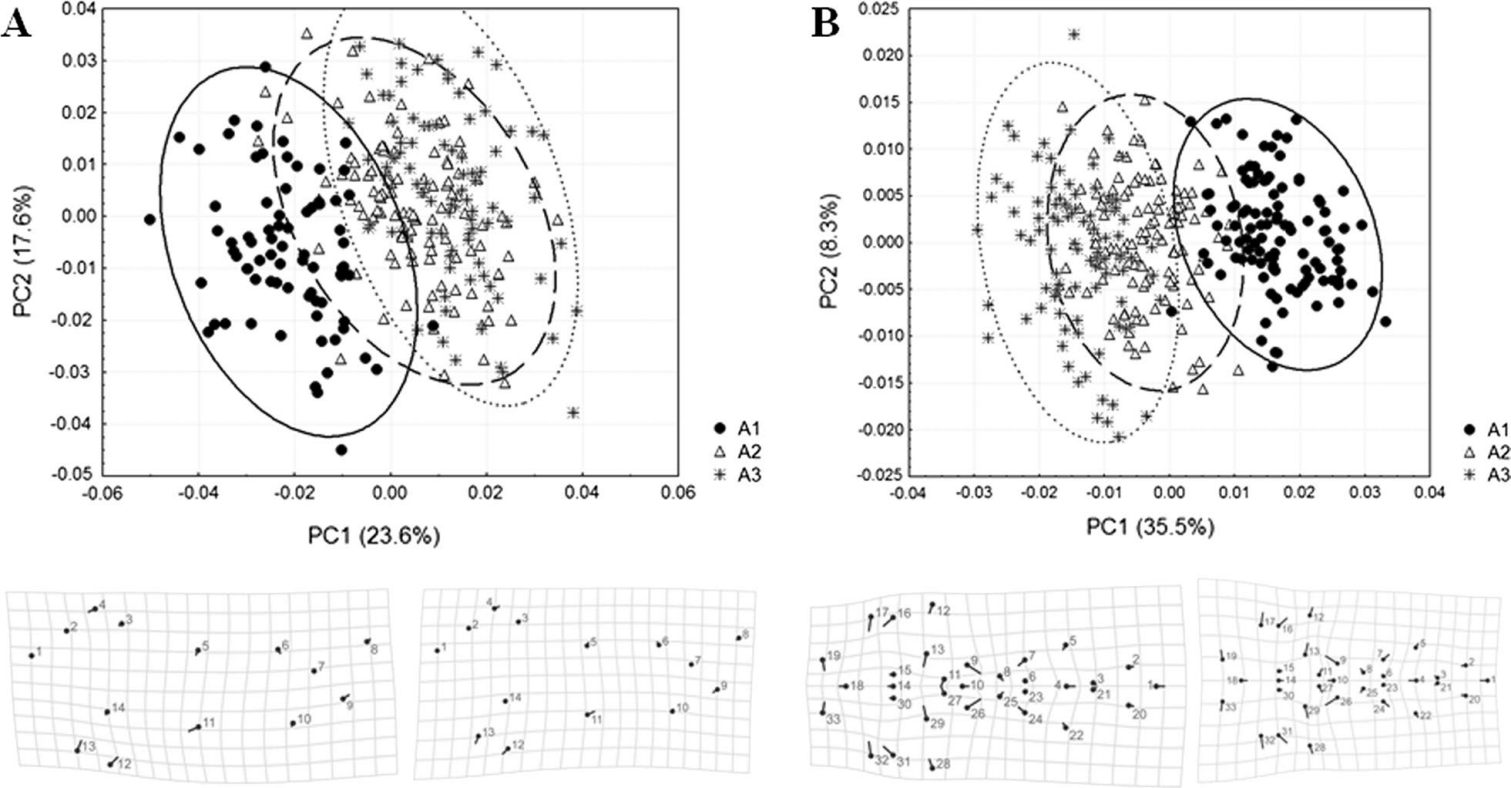
Scatterplots of the first two principal components (PC1 vs. PC2) from the symmetric component of shape variation (individual variation, Ind) for the mandible (**a**) and cranium (**b**). Age categories: A1—first age category, A2—second age category, A3—third age category. Shape changes (magnified 3 times for the cranium) among categories separated along PC1 axis are visualized by TPS deformation grids

All pairwise comparisons of the analyzed age categories reveal statistically significant mean shape differences in symmetric component of shape variation (mandible: Pd (A1 vs. A2) = 0.0280, *P* < 0.0001; Pd (A1 vs. A3) = 0.0362, *P* < 0.0001; Pd (A2 vs. A3) = 0.0109, *P* < 0.0001; cranium: Pd (A1 vs. A2) = 0.0206, *P* < 0.0001; Pd (A1 vs. A3) = 0.0309, *P* < 0.0001; Pd (A2 vs. A3) = 0.0109, *P* < 0.0001). As illustrated by scatterplots of the first two principal components (PC1 and PC2) for symmetric shape variation, age categories separate from each other along PC1 axis, although there is a larger overlap between A2 and A3 categories for the mandible than for the cranium (Fig. 3). Mandibular shape changes between the youngest (A1 category) and two older (A2 and A3 categories) groups are visible almost throughout the whole structure, except the region of condylar process. Additionally, in the youngest yellow-necked mice the posterior part of the mandible (in the regions of angular and coronoid processes) is narrower and the anterior part (alveolar region, i.e. molar and incisor zones) is longer, whereas in older specimens the posterior mandibular part is wider and the alveolar region is shorter (Fig. 3a). Considering cranial shape modifications between the youngest and the oldest (A3 category) individuals, the youngest yellow-necked mice have crania with broader basicranial region, enlarged regions of the palate and foramen magnum, wider and shorter facial region and more laterally expanded zygomatic arches (Fig. 3b).

For both mandible and cranium the patterns of symmetric (Ind) and asymmetric (FA) shape variation is similar between all age categories (Table 3).

**Table 3:** Comparisons of the patterns of covariance for the symmetric (individual variation, Ind) and asymmetric (fluctuating asymmetry, FA) components of shape variation for the mandible and cranium

	Shape component	R	*P*	Shape component	R	*P*
Mandible						
A1 versus A2	Symmetric	0.923	< 0.0001	Asymmetric	0.896	< 0.0001
A1 versus A3	Symmetric	0.925	< 0.0001	Asymmetric	0.821	< 0.0001
A2 versus A3	Symmetric	0.921	< 0.0001	Asymmetric	0.910	< 0.0001
Cranium						
A1 versus A2	Symmetric	0.855	< 0.0001	Asymmetric	0.876	< 0.0001
A1 versus A3	Symmetric	0.829	< 0.0001	Asymmetric	0.879	< 0.0001
A2 versus A3	Symmetric	0.855	< 0.0001	Asymmetric	0.852	< 0.0001

Age categories: A1—first age category, A2—second age category, A3—third age category. R—matrix correlation. *P* values are from matrix permutation tests against the null hypothesis of complete dissimilarity between the respective covariance matrices

### Categories regarding parasitism—size

According to parametric F-tests in ANOVAs of centroid size (CS) (Additional file 4: Table S4), mandibular and cranial size variation among individuals is highly significant in all categories regarding parasitism. Directional asymmetry (DA) and fluctuating asymmetry (FA) for the mandible are also highly significant in all categories, except for DA in P3 category which is significant at *P* = 0.0310. Additional effect of sex is significant in P3 category for the mandible and in P1 and P3 categories for the cranium. Effect of presence of B chromosomes (Bs) is significant only for the mandible within P1 category at *P* = 0.0353. Although significant, DA accounts for a fairly small percentage of the total mandibular size variation (0.22%, 0.14%, 0.13%, and 0.25% in P0, P1, P2, and P3 category, respectively). Kolmogorov–Smirnov tests show that CS asymmetry is normally distributed, indicating the absence of antisymmetry in mandibular size within each category regarding parasitism.

**Fig. 4:**
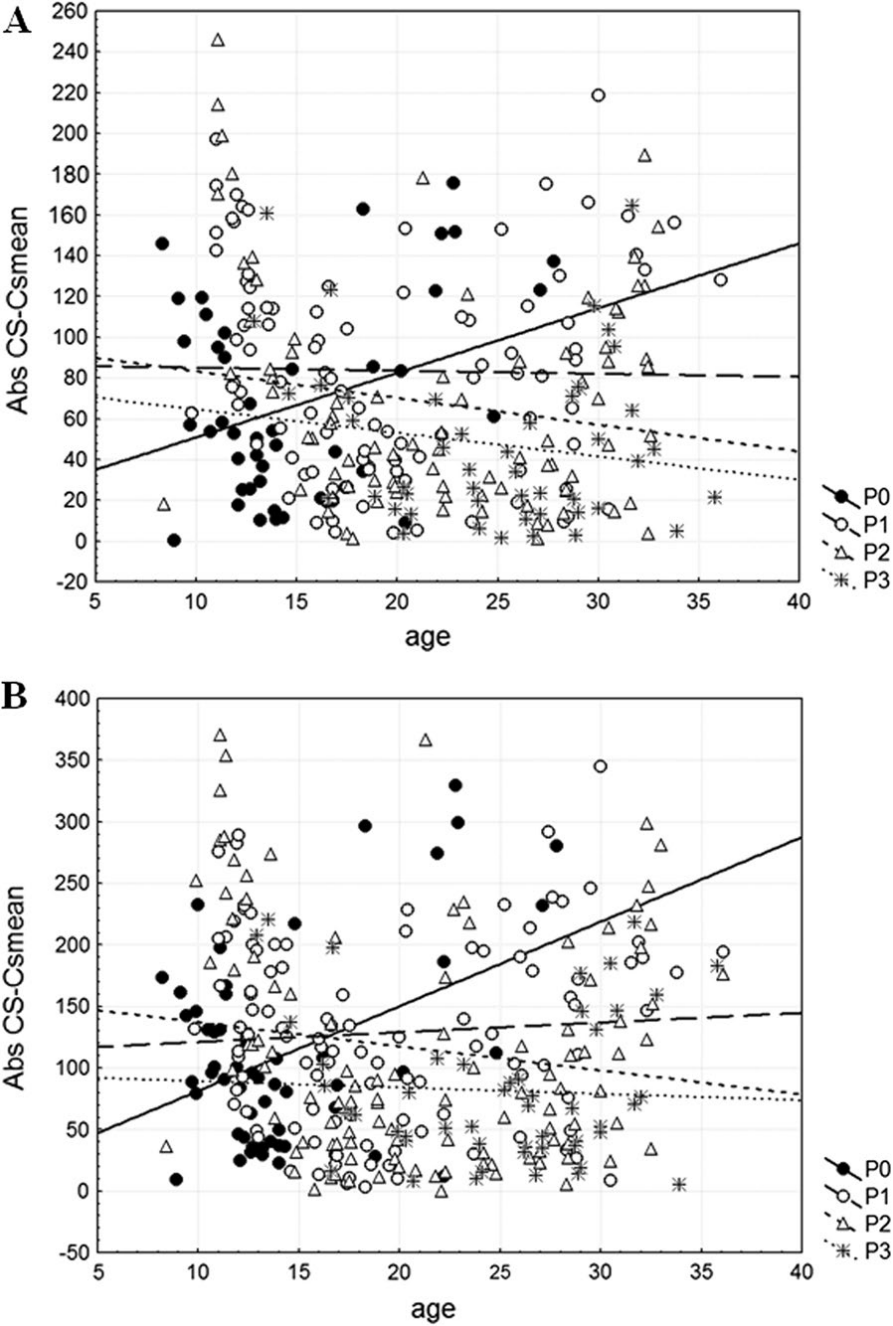
Regressions of individual measures of size variance (absolute size differences) onto dry eye lens mass (age) within each category regarding parasitism (P0—non-parasitized animals, P1—animals parasitized by one nematode species, P2—animals parasitized by two nematode species, P3—animals parasitized by three to five nematode species) for the mandible (**a**) and cranium (**b**)

Both variance of symmetric CS (Var_SymmCS_) for mandibular size and variance of CS (Var_CS_) for cranial size are the highest in P1 category, followed by P2 and P0 categories, whereas the lowest values are observed in P3 category (Table 4). While these differences among categories regarding parasitism in mandibular size variance are not statistically significant (F_3, 271_ = 2.05, *P* = 0.1075), Levene’s test performed on absolute size differences reveals statistically significant differences in cranial size variance (F_3, 316_ = 5.05, *P* = 0.0020). Besides, regressions of absolute size differences on dry eye lens mass show the absence of age-related changes in mandibular and cranial size variance in each category regarding parasitism, except in P0 category (mandible: r = 0.3225, *P* = 0.0288; cranium: r = 0.3992, *P* = 0.0023) where both mandibular and cranial size variance increased with age (Fig. 4).

**Table 4:** Levels of canalization and developmental stability (DS) for the mandible and cranium

	Var_SymmCS_	Var_CS_	Var_shape_	SizeFA10a	ShapeFA10a
Mandible					
P0	6963.96	–	0.00098	10.78	0.00412
P1	9780.19	–	0.00114	13.04	0.00377
P2	7605.12	–	0.00109	6.38	0.00347
P3	3961.81	–	0.00125	15.48	0.00373
Cranium		*			*
P0	–	19,286.69	0.00038	–	0.00132
P1	–	22,648.45	0.00040	–	0.00136
P2	–	22,449.80	0.00041	–	0.00131
P3	–	10,525.37	0.00040	–	0.00134

^*^ Significance level at *P* < 0.01 after Levene’s test

Categories regarding parasitism: P0—non-parasitized animals, P1—animals parasitized by one nematode species, P2—animals parasitized by two nematode species, P3—animals parasitized by three to five nematode species. Var_SymmCS_—mandibular among-individual size variance; Var_CS_—cranial among-individual size variance; Var_shape_—variance in mandibular/cranial shape; SizeFA10a—level of fluctuating asymmetry (FA) for mandibular size; ShapeFA10a—level of fluctuating asymmetry (FA) for mandibular/cranial shape. FA10a = 0.798√MS_sj_-MS_m_ calculated from the values given in Additional files 4 and 5: Tables S4 and S5

For mandibular size the level of FA (SizeFA10a index) is the highest in P3 category, followed by P1 and P0 categories, while the lowest value of SizeFA10a index is observed in P2 category (Table 4). Levene’s test performed on asymmetric CS discloses no statistically significant differences in the level of mandibular size FA among categories regarding parasitism (F_3, 271_ = 1.36, *P* = 0.2540). Likewise, regressions of asymmetric CS versus age indicate the absence of the association of individual measures of size FA with age within each category regarding parasitism (P0 category: r = 0.0834, *P* = 0.5816; P1 category: r = −0.0854, *P* = 0.4082; P2 category: r = −0.0942, *P* = 0.3881; P3 category: r = 0.0209, *P* = 0.8889).

### Categories regarding parasitism—Shape

As presented by parametric F-tests in Procrustes ANOVAs of shape (Additional file 5: Table S5), mandibular and cranial shape variation among individuals, as well as directional asymmetry (DA) and fluctuating asymmetry (FA), are all highly significant in all categories regarding parasitism. Although additional effect of sex is significant in P2 and P3 categories for the mandible and in P2 category for the cranium, it accounts for smaller percentage of the total shape variation than individual variation and FA (mandible—P2 category: Sex = 1.72%; Individual = 81.04%; Ind × Side = 10.66%; mandible—P3 category: Sex = 3.15% Individual = 80.34%; Ind × Side = 10.36%; cranium—P2 category: Sex = 1.31%; Individual = 81.90%; Ind × Side = 10.48%). Additional effect of presence of B chromosomes (Bs) is significant only for the mandible in P1 category where it accounts for smaller percentage of the total shape variation than individual variation and FA (Bs = 1.94%; Individual = 80.43%; Ind × Side = 11.59%). Although significant, DA accounts for a fairly small percentage of the total mandibular and cranial shape variation (mandible: 1.97%, 1.14%, 1.27%, and 1.06% in P0, P1, P2, and P3 category, respectively; cranium: 2.18%, 1.43%, 1.76%, and 2.24% in P0, P1, P2, and P3 category, respectively). In all categories regarding parasitism the visual examinations of the scatter plots for the first five PCs of the vectors of individual signed asymmetries reveal no evidence for clustering of the data points. Additionally, Kolmogorov–Smirnov tests indicate that distributions of each of the five PCs scores don’t show a significant departure from normality. Thus, these asymmetries can be assigned to FAs.

**Fig. 5:**
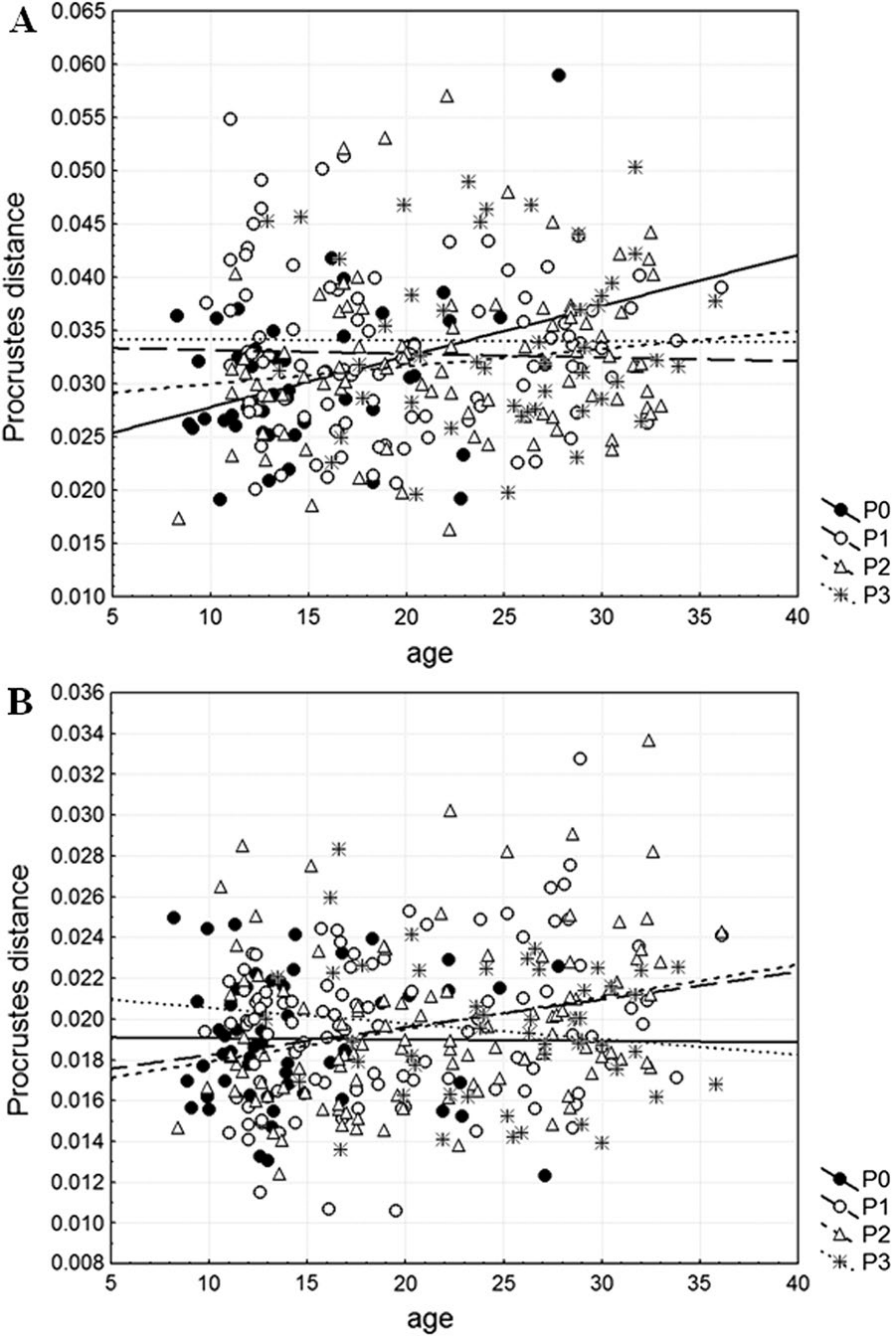
Regressions of individual measures of shape variance (Procrustes distances, Pds) onto dry eye lens mass (age) within each category regarding parasitism (P0—non-parasitized animals, P1—animals parasitized by one nematode species, P2—animals parasitized by two nematode species, P3—animals parasitized by three to five nematode species) for the mandible (**a**) and cranium (**b**)

For the mandible, among-individual shape variance (Var_shape_) is the highest in P3 category, followed by P1 and P2 categories, whereas the lowest value is observed in P0 category (Table 4). Levene’s test performed on Procrustes distances (Pds) reveal no significant differences in mandibular shape variance among categories regarding parasitism (F_3, 271_ = 0.89, *P* = 0.4481). Regressions of Pds on dry eye lens mass show the absence of age-related changes in mandibular shape variance in each category regarding parasitism, except in P0 category (r = 0.3372, *P* = 0.0219) where mandibular shape variance increased with age (Fig. 5a). For the cranium, among-individual shape variance (Var_shape_) is the highest in P2 category, followed by the same values observed in P1 and P3 categories, whereas the lowest value is observed in P0 category (Table 4). Levene’s test performed on Pds reveal no significant differences in cranial shape variance among categories regarding parasitism (F_3, 316_ = 0.82, *P* = 0.4862). Regressions of Pds on dry eye lens mass show the absence of age-related changes in cranial shape variance in P0 and P3 categories, but its presence in P1 (r = 0.2449, *P* = 0.0114) and P2 (r = 0.2856, *P* = 0.0027) categories where cranial shape variance increased with age (Fig. 5b).

**Fig. 6:**
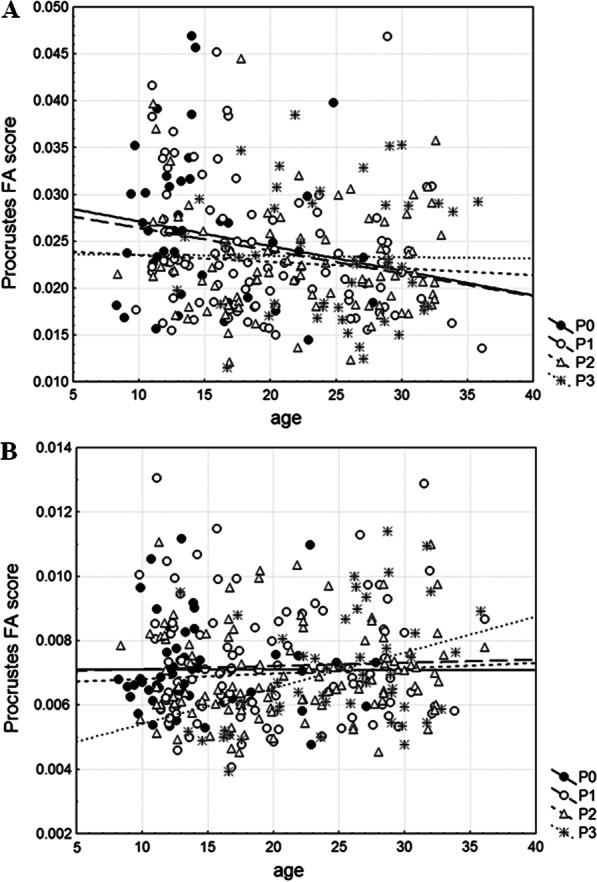
Regressions of individual measures of shape fluctuating asymmetry (Procrustes FA scores) onto dry eye lens mass (age) within each category regarding parasitism (P0—non-parasitized animals, P1—animals parasitized by one nematode species, P2—animals parasitized by two nematode species, P3—animals parasitized by three to five nematode species) for the mandible (**a**) and cranium (**b**)

For mandibular shape the level of FA (ShapeFA10a index) is the highest in P0 category, followed by P1 and P3 categories, whereas the lowest value is observed in P2 category (Table 4). Levene’s test performed on shape FA scores discloses no significant differences in the level of mandibular shape FA among categories regarding parasitism (F_3, 271_ = 1.89, *P* = 0.1316). Regressions of mandibular Procrustes FA scores versus age are statistically insignificant within each category regarding parasitism, except in P1 category (r = −0.2317, *P* = 0.0231) where shape FA scores decreased with age (Fig. 6a). For cranial shape the level of FA (ShapeFA10a index) is the highest in P1 category, followed by P3 and P0 categories, whereas the lowest value is in P2 category (Table 4). Levene’s test performed on shape FA scores reveals statistically significant differences in the level of cranial shape FA among categories regarding parasitism (F_3, 316_ = 4.26, *P* = 0.0057). Regressions of cranial Procrustes FA scores versus age are statistically insignificant within each category regarding parasitism, except in P3 category (r = 0.3608, *P* = 0.0101) where shape FA scores increased with age (Fig. 6b).

**Fig. 7:**
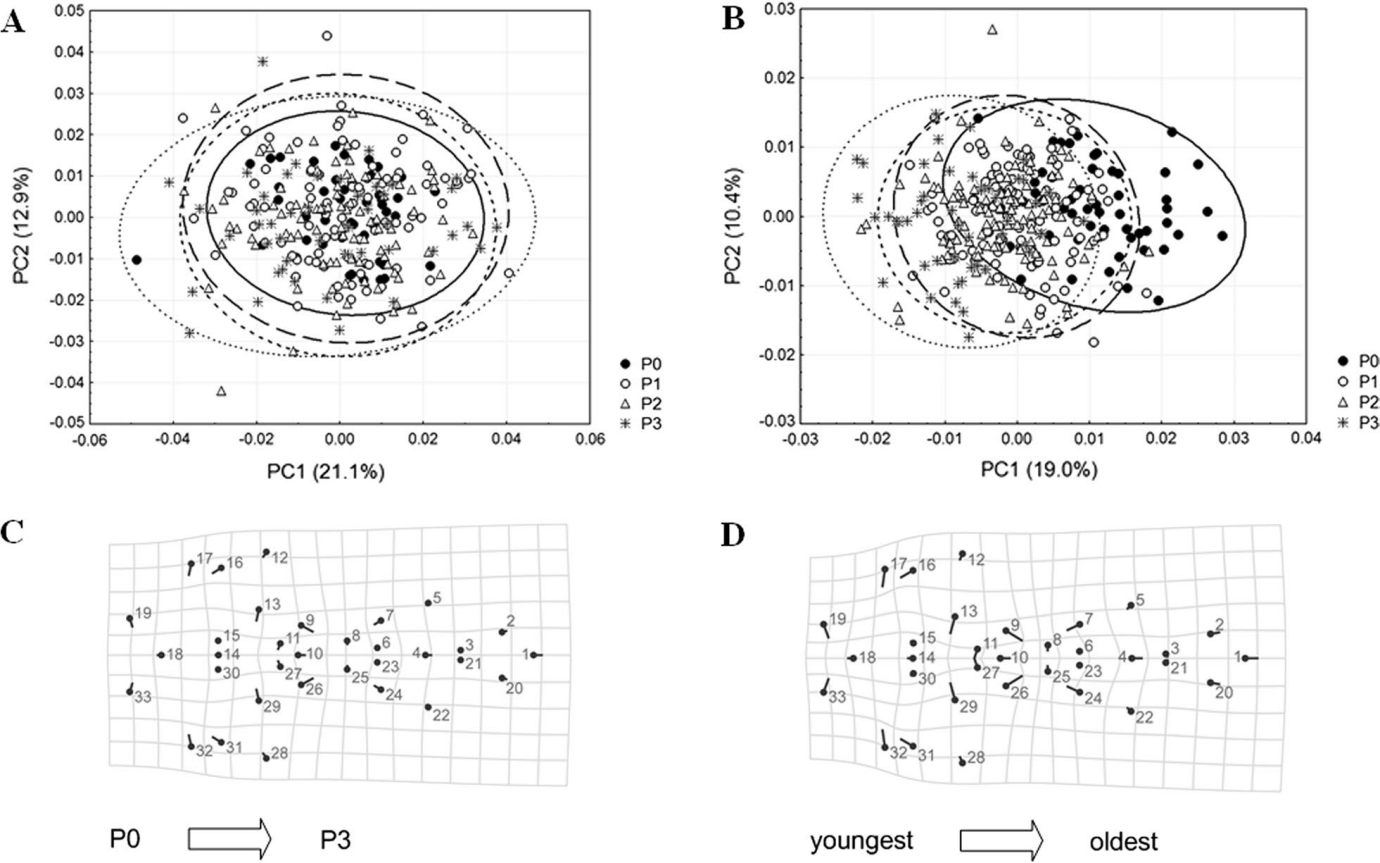
Scatterplots of the first two principal components (PC1 vs. PC2) from the symmetric component of shape variation (individual variation, Ind) for the mandible (**a**) and cranium (**b**). Categories regarding parasitism: P0—non-parasitized animals, P1—animals parasitized by one nematode species, P2—animals parasitized by two nematode species, P3—animals parasitized by three to five nematode species. Cranial shape changes (magnified 3 times and visualized by TPS deformation grids) observed from discriminant function analysis (DFA) between P0 and P3 categories (**c**) and multivariate regression of their shape variables onto dry eye lens mass (**d**)

All pairwise comparisons of the analyzed categories regarding parasitism, except P1 vs. P2 (Pd = 0.0076, *P* = 0.0123; non-significant after Bonferroni correction) and P2 vs. P3 (Pd = 0.0069, *P* = 0.2240) for the mandible and P1 vs. P2 for the cranium (Pd = 0.0032, *P* = 0.1627), show statistically significant mean shape differences in symmetric component of shape variation (mandible: Pd (P0 vs. P1) = 0.0131, *P* < 0.0001; Pd (P0 vs. P2) = 0.0175, *P* < 0.0001; Pd (P0 vs. P3) = 0.0221, *P* < 0.0001; Pd (P1 vs. P3) = 0.0122, *P* = 0.0001; cranium: Pd (P0 vs. P1) = 0.0119, *P* < 0.0001; Pd (P0 vs. P2) = 0.0136, *P* < 0.0001; Pd (P0 vs. P3) = 0.0199, *P* < 0.0001; Pd (P1 vs. P3) = 0.0086 *P* < 0.0001; Pd (P2 vs. P3) = 0.0069, *P* < 0.0001). Scatterplots of the first two principal components (PC1 and PC2) for symmetric shape variation, demonstrate that for the mandible there is a complete overlap of specimens belonging to four categories regarding parasitism (Fig. 7a), while for the cranium PC1 axis tends to separate non-parasitized (P0 category) from the animals parasitized by three to five nematode species (P3 category) (Fig. 7b). However, discriminant function analysis (DFA) between P0 and P3 categories and multivariate regression of their cranial shape variables onto dry eye lens mass reveal similar shape changes (Fig. 7c, d), indicating that variation in the symmetric component of cranial shape variation detected along P1 axis between P0 and P3 categories (Fig. 7b) is age-related.

For both mandible and cranium the patterns of symmetric (Ind) and asymmetric (FA) shape variation is similar between all categories regarding parasitism (Table 5).

**Table 5:** Comparisons of the patterns of covariance for the symmetric (individual variation, Ind) and asymmetric (fluctuating asymmetry, FA) components of shape variation for the mandible and cranium

	Shape component	R	*P*	Shape component	R	*P*
Mandible						
P0 versus P1	Symmetric	0.899	< 0.0001	Asymmetric	0.863	< 0.0001
P0 versus P2	Symmetric	0.890	< 0.0001	Asymmetric	0.846	< 0.0001
P0 versus P3	Symmetric	0.831	< 0.0001	Asymmetric	0.761	< 0.0001
P1 versus P2	Symmetric	0.946	< 0.0001	Asymmetric	0.906	< 0.0001
P1 versus P3	Symmetric	0.911	< 0.0001	Asymmetric	0.872	< 0.0001
P2 versus P3	Symmetric	0.899	< 0.0001	Asymmetric	0.899	< 0.0001
Cranium						
P0 versus P1	Symmetric	0.819	< 0.0001	Asymmetric	0.870	< 0.0001
P0 versus P2	Symmetric	0.841	< 0.0001	Asymmetric	0.837	< 0.0001
P0 versus P3	Symmetric	0.804	< 0.0001	Asymmetric	0.824	< 0.0001
P1 versus P2	Symmetric	0.890	< 0.0001	Asymmetric	0.897	< 0.0001
P1 versus P3	Symmetric	0.836	< 0.0001	Asymmetric	0.859	< 0.0001
P2 versus P3	Symmetric	0.844	< 0.0001	Asymmetric	0.852	< 0.0001

Categories regarding parasitism: P0—non-parasitized animals, P1—animals parasitized by one nematode species, P2—animals parasitized by two nematode species, P3—animals parasitized by three to five nematode species. R—matrix correlation. *P* values are from matrix permutation tests against the null hypothesis of complete dissimilarity between the respective covariance matrices

## Discussion

### Age-related canalization and developmental stability

In contrast to our prediction, mandibular and cranial size variance among *Apodemus flavicollis* individuals does not decrease with age, indicating the absence of age-related changes in the level of canalization for mandibular and cranial size. This discrepancy from the previous examinations of laboratory-reared [23, 25, 35], as well as rodents from the wild [49], that documented statistically significant reduction in skull size variance among individuals in older groups of specimens, is probably due to the facts that our study does not include juveniles and that growth is asymptotic in the previous studies.

We detected statistically significant increase of both mandibular and cranial shape variance among *A. flavicollis* individuals with age, i.e. age-related decline of the level of canalization for mandibular and cranial shape. This result contradicts our prediction, as well as earlier findings of statistically significant reduction in skull shape variance among individuals in older groups of specimens in laboratory-raised rodents [23, 36, 37]. On the other hand, it is in agreement with findings by Willmore et al. [25]. Inspecting the effects of developmental and functional interactions on mouse cranial variability through late ontogeny, Willmore et al. [25] surveyed random-bred mouse skulls aged 35, 90, and 150 days and found that the youngest group (35 days old mice) was characterized by the smallest value of among-individual shape variance for mesoderm and basicranial regions, as well as the whole skull. The highest amounts of mandibular and cranial shape variation among *A. flavicollis* individuals observed in the oldest group, i.e. the reduction in the level of canalization for mandibular and cranial shape with age, could be explained by the fact that animals from natural populations, in contrast to those from laboratories, would be exposed to heterogeneous environmental conditions, and their diet would probably differ greatly from that of lab animals, as suggested by Willmore et al. [25]. Accordingly, increased environmental variation would result in increased phenotypic variation which could possibly create changes in variability through late postnatal ontogeny that are not seen in laboratory samples.

Age-related dynamics of the magnitude of *A. flavicollis* intraindividual mandibular size variation (FA) is not statistically significant. Once again, the absence of differences in the level of DS for mandibular size among age categories may be a consequence of the absence of juveniles in the study sample. However, for mandibular shape, the level of DS increases with age, whereas for cranial shape there are no age-related changes in the level of DS. DS was found to decrease with age postnatally in both macaque and human crania [98]. Nevertheless, Parker and Leamy [99] documented its increase with age in random-bred mouse morphometric characters, while Willmore et al. [25] reported that the degree of FA in random-bred mouse skulls was stable between three age groups (35, 90, and 150 days) for the whole skull, as well as all its regions.

Investigations of laboratory-reared mice and rats [25, 37, 100], as well as studies that have used specimens from wild rodent populations [49, 101], demonstrated that the mean skull shape differed across ontogenetic stages. The values of Procrustes distances for both mandible and cranium of *A. flavicollis* point to the largest differences in the mean shape between A1 and A3, followed by A1 and A2, and the smallest ones between A2 and A3 categories. As evident from the PCA scatterplots of the first two principal components (PC1 and PC2) for symmetric shape variation, PC1 axis separates age categories. As expected, mandibular and cranial shape changes are most visible between the youngest and the oldest age groups, supporting the earlier findings in rodents [25, 37, 49, 100, 101]. Concerning shape differences between the successive age categories, there is a similar degree of overlap for the mandible and cranium between A1 and A2 categories, whereas between A2 and A3 categories there is a greater degree of overlap for the mandible than for the cranium. Mandibular shape changes between the youngest and two older groups, i.e. the wider posterior mandibular part (in the regions of angular and coronoid processes) and the shorter alveolar region (molar and incisor zones) in older *A. flavicollis* specimens, are concordant with the ontogenetic mandibular shape variation in eight rodent species observed by Zelditch et al. [102]. These authors noted the general deepening of the horizontal ramus relative to its length (shortening of the mandible anteriorly), the tremendous deepening of the angular process, and a substantial increase in the width of the coronoid process, as well as a reorientation of that process to be more erect, as the most striking mandibular shape similarity between the postnatal ontogenies of the analyzed species, including cotton rat (*Sigmodon fulviventer*) and house mouse (*Mus musculus domesticus*). Cranial shape changes between the youngest and the oldest groups, i.e. reduced basicranial region, but longer and narrower facial region in older *A. flavicollis* specimens, are in agreement with the common postnatal ontogenetic cranial shape changes in tetrapods [103], while contracted regions of the palate and foramen magnum in older *A. flavicollis* are consistent with findings of postnatal ontogenetic cranial shape variation in the yellow-bellied marmot *Marmota flaviventris* [101] and Martino’s vole *Dinaromys bogdanovi* [49].

Contrary to our prediction, there is no age-related dynamics of covariance structure among *A. flavicollis* individuals. Likewise, we detected no ontogenetic dynamics of covariance pattern within *A. flavicollis* individuals. Analyzing covariance structures from age to age in two rodent species, cotton rats (*S. fulviventer*) and house mice (*M. m. domesticus*), Zelditch et al. [24] also assessed the overall similarity of covariance matrices by using matrix correlation, but found significant differences in structure of variation, measured by the angles between the subspaces encompassing 80% of the variation. Moreover, assessing the effect of prenatal nutritional stress on canalization and DS of the laboratory mouse fetal skull, Gonzalez et al. [30] found that the pattern of shape asymmetry was affected by the environmental perturbation, by estimating the angles between the first PCs. Therefore, besides biological, methodological issues cannot be omitted as a possible reason for the failure to detect ontogenetic dynamics of covariance structure both among and within *A. flavicollis* individuals.

### Nematode parasitism-related canalization and developmental stability

Regarding the relationship of nematode parasitism with canalization for mandibular and cranial size and shape in *A. flavicollis*, we predicted the highest level of canalization in non-parasitized animals (P0 category). Disagreeing with our prediction, the most parasitized category (P3) was characterized by the highest level of canalization observed for mandibular and cranial size. Even though in P0 category the level of canalization for both mandibular and cranial shape is the highest as we expected, we observed no statistically significant differences in mandibular and cranial shape variance among analyzed categories regarding parasitism. In P0 category we detected statistically significant increase of mandibular size and shape variance and cranial size variance with age, indicating age-related decline of the level of canalization in non-parasitized animals. Age-related reduction in the level of canalization for cranial shape is detected in P1 and P2 categories. As concluded previously, age-related reduction in the level of canalization in wild compared to laboratory-reared animals could be explained by differences in environments animals from natural and captive habitats experience. Heterogeneous environmental conditions, particularly diverse diet, would result in increased phenotypic variation through late postnatal ontogeny in wild animals. As infections by parasites may be associated with host diet choice [104], differences in age-related dynamics of the level of canalization between non-parasitized and parasitized animals, as well as between yellow-necked mice parasitized by different number of nematode species, may also be related to potential diet differences between them.

Considering the magnitude of mandibular size and shape variation within *A. flavicollis* individuals (FA), inconsistent with our prediction, the level of DS for both mandibular size and shape is the highest in P2 category, while the level of DS for mandibular shape is the lowest in P0 category. These differences in the level of mandibular size and shape FA among categories regarding parasitism are not statistically significant. We found statistically significant differences in the level of FA for cranial shape among analyzed categories regarding parasitism. However, contradictory to our prediction, the level of DS for cranial shape is the highest in P2, followed by P0 and P3 category, and the lowest in P1 category. We detected statistically significant association of individual measures of mandibular and cranial shape FA with age. Namely, individual measures of mandibular and cranial shape FA significantly decreased with age for the mandible in the less parasitized category (P1) and increased for the cranium in the most parasitized category (P3). Observed ontogenetic trend in mandibular shape FA in P1 category correspond to that identified within the whole sample for age categories, i.e. the level of DS for mandibular shape increases with age. If parasites directly act on the developmental stability of their hosts by imposing on them a metabolic cost during their ontogeny [52, 56], it is predicted that host developmental instability should be directly related to parasite virulence, parasite load, or both [54, 56–58]. Therefore, it seems likely that the detected relationship between cranial shape DS and nematode infections in *A. flavicollis* is caused by parasitic disruption of host development and their direct effect on host DS, i.e. by the third reason proposed by Møller [52]. It is also possible that yellow-necked mice with higher levels of cranial shape FA will more often be restricted to poor environments with elevated risk of encountering parasites [14, 52, 54, 55] or they may be more susceptible to parasitism [52–54]. Body condition is related to an animal’s health, quality or vigour [105] and is indicative of environmental stress [106]. Due to the fact that we found no difference in the body condition indices (BCI) among categories regarding parasitism, we can eliminate second reason proposed by Møller [52]. Since parasites can be a cause of asymmetry, and asymmetry can be an indicator of susceptibility to parasitism [17], it would be unwarranted to single out any of these two reasons suggested by Møller [52].

Discrepancy of the results related to the model systems (mandible/cranium) could be explained by possible trait-specific effects of environmental perturbations and developmental noise acting upon the level of canalization and DS, respectively. Different traits might be affected differently by the same conditions owing to different growth curves or different functions of the traits [107]. Moreover, different morphological traits may vary in their susceptibility to the effects of parasitism [52]. For example, in male reindeer Markusson and Folstad [108] reported that the parasite index was positively correlated to asymmetry in antler length, weight and volume, but not to asymmetry in number of tines or asymmetry in jaw length.

Contradicting our prediction, infections by intestinal nematodes are not associated with mandibular and cranial shape variation among *A. flavicollis* individuals. Likewise, we reported the absence of statistically significant differences in the pattern of symmetric and asymmetric shape variation between non-parasitized and parasitized *A. flavicollis* specimens, as well as between yellow-necked mice parasitized by different number of nematode species, for both mandible and cranium. However, as we previously concluded, besides biological, methodological issues cannot be omitted as a possible reason for the failure to detect differences in covariance structures.

On a final note, advantages of laboratory-based studies over those that include animals from wild populations are obvious. Studies of experimental developmental biology use animals characterized by low levels of genetic variation, such as inbred mice and rats, and control environmental factors, whereas in natural populations multiple genetic and environmental determinants act on magnitude and pattern of morphological variation. On the other hand, development of a population developmental biology approach based on developmental stability study in natural populations is recognized by Zakharov et al. [4] as particularly promising for solving various problems in a practice of population studies as well as for obtaining information on the state of homeorhesis in nature for developmental biology. Finally, to make reasonable predictions about how a covariance structure among and/or within individuals, i.e. pattern of canalization and/or DS, might respond to some hypothetical perturbation or change, an alternative approach, as advocated by Hallgrímsson et al. [109], is to combine the analyses of natural populations with model organisms in which the source of covariance is known to some extent or can be controlled.

## Conclusions

Our age-related results partly agree with previous findings in rodents. We observed the absence of age-related changes in the levels of canalization for mandibular and cranial size, but found age-related decline of the level of canalization for mandibular and cranial shape and age-related increase of the level of DS for mandibular shape. We detected mandibular and cranial shape changes during postnatal ontogeny, but revealed no age-related dynamics of mandibular and cranial covariance structure among individuals. However, no rodent study so far has explored age-related changes in the magnitude of FA for mandibular size or mandibular and cranial FA covariance structure. We found the absence of age-related changes in the level of DS for mandibular size. Likewise, we detected no age-related dynamics of mandibular and cranial FA covariance structure.

This is the first study dealing with the nematode parasitism-related canalization and DS in rodents. We found that categories regarding parasitism differ in the level of canalization for cranial size and the level of DS for cranial shape. We observed differences in age-related dynamics of the level of canalization between non-parasitized and parasitized animals, as well as between yellow-necked mice parasitized by different number of nematode species. As infections by parasites may be associated with host diet choice, these differences between categories regarding parasitism may be related to potential diet differences between them. Individual measures of mandibular and cranial shape FA decreased with age for the mandible in the less parasitized category and increased for the cranium in the most parasitized category. Detected relationship between cranial shape DS and nematode infections in *A. flavicollis* may be caused by parasitic disruption of host development and their direct effect on host DS or the most parasitized animals may be more susceptible to parasitism. Discrepancy of the results related to the model systems (mandible/cranium) could be explained by trait-specific effects of environmental perturbations and developmental noise acting upon the level of canalization and DS, respectively. Mandibular and cranial shape variation and covariance structure among and within individuals are not affected by the nematode parasitism.

Overall, additional studies concerning both age- and nematode parasitism-related canalization and DS of rodent skulls, particularly those from natural populations, are required before drawing some general conclusions.

## Supplementary Information

**Additional file 1**. Table S1 Anatomical definitions of landmarks recorded on the labial view of the mandible and the ventral surface of the cranium of the yellow-necked field mouse (*Apodemus flavicollis*)

**Additional file 2**. Table S2 ANOVAs of centroid size (CS). % total—percentage of the total size variation. Age categories: A1—first age category, A2—second age category, A3—third age category

**Additional file 3**. Table S3 Procrustes ANOVAs of shape. % total—percentage of the total shape variation. Age categories: A1—first age category, A2—second age category, A3—third age category

**Additional file 4**. Table S4 ANOVAs of centroid size (CS). % total—percentage of the total size variation. Categories regarding parasitism: P0—non-parasitized animals, P1—animals parasitized by one nematode species, P2—animals parasitized by two nematode species, P3—animals parasitized by three to five nematode species

**Additional file 5**. Table S5 Procrustes ANOVAs of shape. % total—percentage of the total shape variation. Categories regarding parasitism: P0—non-parasitized animals, P1—animals parasitized by one nematode species, P2—animals parasitized by two nematode species, P3—animals parasitized by three to five nematode species

## Data Availability

The datasets used and/or analyzed during the current study are available from the corresponding author on reasonable request.

## Abbreviations

A1: First age category
A2: Second age category
A3: Third age category
ANOVA: Analysis of Variance
Bs: B chromosomes
CS: Centroid size
DA: Directional asymmetry
DFA: Discriminant Function Analysis
DS: Developmental stability
FA: Fluctuating asymmetry/Within-individual variation/Asymmetric shape variation
GPA: Generalized Procrustes Analysis
Ind: Among-individual variation/Symmetric shape variation
P0: Non-parasitized animals
P1: Animals parasitized by one nematode species
P2: Animals parasitized by two nematode species
P3: Animals parasitized by three to five nematode species
PCA: Principal Component Analysis
PC1: First principal component
Pd: Procrustes distance
