# Supplementary material for: Canalization and developmental stability of the yellow-necked mouse (Apodemus flavicollis) mandible and cranium related to age and nematode parasitism
Source: Front Zool. 2021 Oct 24;18:55. doi: 10.1186/s12983-021-00439-4 (PMC8543932; doi:10.1186/s12983-021-00439-4)
Supplement: Supplementary file 1 — Additional file 1. Table S1 Anatomical definitions of landmarks recorded on the labial view of the mandible and the ventral surface of the cranium of the yellow-necked field mouse (Apodemus flavicollis) [file 12983_2021_439_MOESM1_ESM.docx]

**Additional file 1: Table S1** Anatomical definitions of landmarks recorded on the labial view of the mandible and the ventral surface of the cranium of the yellow-necked field mouse (*Apodemus flavicollis*)

| **mandible** | |  | **cranium** | |
| --- | --- | --- | --- | --- |
| **1** | Posterior tip of the condyle |  | **1** | Anterior extremity of the suture between the nasals |
| **2** | Anterior tip of the condyle |  | **2, 20** | Lateral margin of incisive alveolus where it intersects outline of the skull in photographic plane |
| **3** | Maximum of curvature between coronoid and condylar processes |  | **3, 21** | Anterior-most point of incisive foramen |
| **4** | Tip of the coronoid process |  | **4** | Suture between the vomerine portion of the premaxillary and maxillary in the incisive foramen |
| **5** | Posterior intersection of the molar tooth row with the coronoid surface |  | **5, 22** | Anterior extremity of the zygomatic plate |
| **6** | Anterior edge of the molar tooth row |  | **6, 23** | Posterior-most point of incisive foramen |
| **7** | Extreme of the diastema invagination |  | **7, 24** | Intersection between the anterior end of the premolar and maxillary |
| **8** | Antero-dorsal border of the incisive alveolus |  | **8, 25** | Anterior border of the posterior palatine foramen |
| **9** | Antero-ventral border of the incisive alveolus |  | **9, 26** | Intersection between the posterior end of the 3rd upper molar (M3) and maxillary |
| **10** | Anterior-most point on the baseline perpendicular to the landmark 6 |  | **10** | Back of palatine |
| **11** | Dorsal-most point on the ventral border of the mandible |  | **11, 27** | Lateral-most point in the suture between the presphenoid and basisphenoid |
| **12** | Ventral-most point on the ventral border of angular process |  | **12, 28** | Anterior region of the squamosal zygomatic process where it joins the zygomatic arch |
| **13** | Tip of the angular process |  | **13, 29** | Anterior-most point of foramen ovale |
| **14** | Maximum of curvature on the curve between the condylar and angular processes |  | **14** | Midpoint of basisphenoid-basioccipital suture |
|  |  |  | **15, 30** | Point where the suture between the basisphenoid and basioccipital contacts the tympanic bulla |
|  |  |  | **16, 31** | Anterior tip of the external auditory meatus |
|  |  |  | **17, 32** | Posterior tip of the external auditory meatus |
|  |  |  | **18** | Anterior-most point of the foramen magnum |
|  |  |  | **19, 33** | Lateral tip of the occipital condyle |
