# Supplementary material for: Canalization and developmental stability of the yellow-necked mouse (Apodemus flavicollis) mandible and cranium related to age and nematode parasitism
Source: Front Zool. 2021 Oct 24;18:55. doi: 10.1186/s12983-021-00439-4 (PMC8543932; doi:10.1186/s12983-021-00439-4)
Supplement: Supplementary file 2 — Additional file 2. Table S2 ANOVAs of centroid size (CS). % total—percentage of the total size variation. Age categories: A1—first age category, A2—second age category, A3—third age category [file 12983_2021_439_MOESM2_ESM.docx]

**Additional file 2: Table S2** ANOVAs of centroid size (CS). % total – percentage of the total size variation. Age categories: A1 – first age category, A2 – second age category, A3 – third age category

|  | Effect | MS | df | F | P | % total |
| --- | --- | --- | --- | --- | --- | --- |
| Mandible |  |  |  |  |  |  |
| A1 | Sex | 33.33 | 1 | 0.00 | 0.9554 | 0.00 |
|  | B chromosomes | 5243.15 | 1 | 0.50 | 0.4834 | 0.66 |
|  | Individual | 10567.64 | 74 | 143.62 | <0.0001 | 97.92 |
|  | Side | 3203.73 | 1 | 43.54 | <0.0001 | 0.40 |
|  | Ind x Side | 73.58 | 76 | 4.53 | <0.0001 | 0.70 |
|  | Error | 16.24 | 154 |  |  | 0.31 |
|  |  |  |  |  |  |  |
| A2 | Sex | 110997.77 | 1 | 8.93 | 0.0035 | 7.43 |
|  | B chromosomes | 19642.79 | 1 | 1.58 | 0.2114 | 1.31 |
|  | Individual | 12428.09 | 107 | 96.32 | <0.0001 | 88.96 |
|  | Side | 3956.10 | 1 | 30.66 | <0.0001 | 0.26 |
|  | Ind x Side | 129.04 | 109 | 1.73 | 0.0003 | 0.94 |
|  | Error | 74.49 | 220 |  |  | 1.10 |
|  |  |  |  |  |  |  |
| A3 | Sex | 137617.81 | 1 | 13.70 | 0.0004 | 13.56 |
|  | B chromosomes | 6194.24 | 1 | 0.62 | 0.4345 | 0.61 |
|  | Individual | 10045.10 | 85 | 81.78 | <0.0001 | 84.11 |
|  | Side | 4973.57 | 1 | 40.49 | <0.0001 | 0.49 |
|  | Ind x Side | 122.84 | 87 | 11.90 | <0.0001 | 1.05 |
|  | Error | 10.32 | 176 |  |  | 0.18 |
| Cranium |  |  |  |  |  |  |
| A1 | Sex | 85.25 | 1 | 0.01 | 0.9411 | 0.01 |
|  | B chromosomes | 4081.34 | 1 | 0.26 | 0.6094 | 0.25 |
|  | Individual | 15535.94 | 104 | 7472.07 | <0.0001 | 99.73 |
|  | Error | 2.08 | 107 |  |  | 0.01 |
|  |  |  |  |  |  |  |
| A2 | Sex | 224912.29 | 1 | 12.71 | 0.0005 | 9.87 |
|  | B chromosomes | 2247.71 | 1 | 0.13 | 0.7222 | 0.10 |
|  | Individual | 17691.31 | 116 | 6522.58 | <0.0001 | 90.02 |
|  | Error | 2.71 | 119 |  |  | 0.01 |
|  |  |  |  |  |  |  |
| A3 | Sex | 233961.04 | 1 | 18.93 | <0.0001 | 16.92 |
|  | B chromosomes | 24359.95 | 1 | 1.97 | 0.1637 | 1.76 |
|  | Individual | 12356.64 | 91 | 3153.90 | <0.0001 | 81.30 |
|  | Error | 3.92 | 94 |  |  | 0.03 |
