# Supplementary material for: Canalization and developmental stability of the yellow-necked mouse (Apodemus flavicollis) mandible and cranium related to age and nematode parasitism
Source: Front Zool. 2021 Oct 24;18:55. doi: 10.1186/s12983-021-00439-4 (PMC8543932; doi:10.1186/s12983-021-00439-4)
Supplement: Supplementary file 3 — Additional file 3. Table S3 Procrustes ANOVAs of shape. % total—percentage of the total shape variation. Age categories: A1—first age category, A2—second age category, A3—third age category [file 12983_2021_439_MOESM3_ESM.docx]

**Additional file 3: Table S3** Procrustes ANOVAs of shape. % total – percentage of the total shape variation. Age categories: A1 – first age category, A2 – second age category, A3 – third age category

|  | Effect | MS | df | F | P | % total |
| --- | --- | --- | --- | --- | --- | --- |
| Mandible |  |  |  |  |  |  |
| A1 | Sex | 0.0003394 | 24 | 2.04 | 0.0022 | 2.10 |
|  | B chromosomes | 0.0002654 | 24 | 1.59 | 0.0342 | 1.64 |
|  | Individual | 0.0001666 | 1776 | 5.33 | <0.0001 | 76.31 |
|  | Side | 0.0001715 | 24 | 5.49 | <0.0001 | 1.06 |
|  | Ind x Side | 0.0000313 | 1824 | 7.15 | <0.0001 | 14.71 |
|  | Error | 0.0000044 | 3696 |  |  | 4.17 |
|  |  |  |  |  |  |  |
| A2 | Sex | 0.0001950 | 24 | 1.07 | 0.3680 | 0.82 |
|  | B chromosomes | 0.0001276 | 24 | 0.70 | 0.8552 | 0.54 |
|  | Individual | 0.0001819 | 2568 | 7.49 | <0.0001 | 82.10 |
|  | Side | 0.0003334 | 24 | 13.73 | <0.0001 | 1.41 |
|  | Ind x Side | 0.0000243 | 2616 | 5.66 | <0.0001 | 11.16 |
|  | Error | 0.0000043 | 5280 |  |  | 3.98 |
|  |  |  |  |  |  |  |
| A3 | Sex | 0.0003481 | 24 | 1.81 | 0.0097 | 1.76 |
|  | B chromosomes | 0.0001493 | 24 | 0.77 | 0.7730 | 0.75 |
|  | Individual | 0.0001929 | 2040 | 8.57 | <0.0001 | 82.77 |
|  | Side | 0.0002920 | 24 | 12.98 | <0.0001 | 1.47 |
|  | Ind x Side | 0.0000225 | 2088 | 5.93 | <0.0001 | 9.88 |
|  | Error | 0.0000038 | 4224 |  |  | 3.37 |
| Cranium |  |  |  |  |  |  |
| A1 | Sex | 0.0000282 | 31 | 1.20 | 0.2079 | 0.92 |
|  | B chromosomes | 0.0000258 | 31 | 1.09 | 0.3294 | 0.84 |
|  | Individual | 0.0000236 | 3224 | 6.81 | <0.0001 | 80.00 |
|  | Side | 0.0000581 | 31 | 16.79 | <0.0001 | 1.90 |
|  | Ind x Side | 0.0000035 | 3286 | 5.52 | <0.0001 | 11.97 |
|  | Error | 0.0000006 | 6634 |  |  | 4.37 |
|  |  |  |  |  |  |  |
| A2 | Sex | 0.0000182 | 31 | 0.74 | 0.8468 | 0.53 |
|  | B chromosomes | 0.0000298 | 31 | 1.22 | 0.1902 | 0.86 |
|  | Individual | 0.0000245 | 3596 | 7.71 | <0.0001 | 82.23 |
|  | Side | 0.0000558 | 31 | 17.58 | <0.0001 | 1.62 |
|  | Ind x Side | 0.0000032 | 3658 | 5.58 | <0.0001 | 10.84 |
|  | Error | 0.0000006 | 7378 |  |  | 3.92 |
|  |  |  |  |  |  |  |
| A3 | Sex | 0.0000430 | 31 | 1.56 | 0.0244 | 1.39 |
|  | B chromosomes | 0.0000364 | 31 | 1.33 | 0.1081 | 1.18 |
|  | Individual | 0.0000275 | 2821 | 7.41 | <0.0001 | 80.70 |
|  | Side | 0.0000567 | 31 | 15.29 | <0.0001 | 1.83 |
|  | Ind x Side | 0.0000037 | 2883 | 5.98 | <0.0001 | 11.14 |
|  | Error | 0.0000006 | 5828 |  |  | 3.77 |
