# Supplementary material for: Canalization and developmental stability of the yellow-necked mouse (Apodemus flavicollis) mandible and cranium related to age and nematode parasitism
Source: Front Zool. 2021 Oct 24;18:55. doi: 10.1186/s12983-021-00439-4 (PMC8543932; doi:10.1186/s12983-021-00439-4)
Supplement: Supplementary file 4 — Additional file 4. Table S4 ANOVAs of centroid size (CS). % total—percentage of the total size variation. Categories regarding parasitism: P0—non-parasitized animals, P1—animals parasitized by one nematode species, P2—animals parasitized by two nematode species, P3—animals parasitized by three to five nematode species [file 12983_2021_439_MOESM4_ESM.docx]

**Additional file 4: Table S4** ANOVAs of centroid size (CS). % total – percentage of the total size variation. Categories regarding parasitism: P0 – non-parasitized animals, P1 – animals parasitized by one nematode species, P2 – animals parasitized by two nematode species, P3 – animals parasitized by three to five nematode species

|  | Effect | MS | df | F | P | % total |
| --- | --- | --- | --- | --- | --- | --- |
| Mandible |  |  |  |  |  |  |
| P0 | Sex | 1856.31 | 1 | 0.06 | 0.8018 | 0.15 |
|  | B chromosomes | 25.96 | 1 | 0.00 | 0.9763 | 0.00 |
|  | Individual | 29107.71 | 43 | 152.71 | <0.0001 | 98.90 |
|  | Side | 2733.52 | 1 | 14.34 | 0.0004 | 0.22 |
|  | Ind x Side | 190.61 | 45 | 23.95 | <0.0001 | 0.68 |
|  | Error | 7.96 | 92 |  |  | 0.06 |
|  |  |  |  |  |  |  |
| P1 | Sex | 39031.27 | 1 | 1.04 | 0.3115 | 1.04 |
|  | B chromosomes | 171928.11 | 1 | 4.56 | 0.0353 | 4.58 |
|  | Individual | 37693.70 | 93 | 132.50 | <0.0001 | 93.42 |
|  | Side | 5349.14 | 1 | 18.80 | <0.0001 | 0.14 |
|  | Ind x Side | 284.48 | 95 | 16.14 | <0.0001 | 0.72 |
|  | Error | 17.62 | 192 |  |  | 0.09 |
|  |  |  |  |  |  |  |
| P2 | Sex | 30865.65 | 1 | 1.01 | 0.3187 | 1.19 |
|  | B chromosomes | 9190.78 | 1 | 0.30 | 0.5856 | 0.35 |
|  | Individual | 30670.92 | 83 | 425.46 | <0.0001 | 98.04 |
|  | Side | 3282.27 | 1 | 45.53 | <0.0001 | 0.13 |
|  | Ind x Side | 72.09 | 85 | 8.77 | <0.0001 | 0.24 |
|  | Error | 8.22 | 172 |  |  | 0.05 |
|  |  |  |  |  |  |  |
| P3 | Sex | 81501.19 | 1 | 5.62 | 0.0222 | 10.87 |
|  | B chromosomes | 9380.19 | 1 | 0.65 | 0.4256 | 1.25 |
|  | Individual | 14502.08 | 44 | 37.62 | <0.0001 | 85.14 |
|  | Side | 1909.31 | 1 | 4.95 | 0.0310 | 0.25 |
|  | Ind x Side | 385.46 | 46 | 42.20 | <0.0001 | 2.37 |
|  | Error | 9.14 | 94 |  |  | 0.11 |
| Cranium |  |  |  |  |  |  |
| P0 | Sex | 1267.23 | 1 | 0.03 | 0.8591 | 0.06 |
|  | B chromosomes | 8580.68 | 1 | 0.22 | 0.6445 | 0.40 |
|  | Individual | 39843.16 | 53 | 17863.97 | <0.0001 | 99.53 |
|  | Error | 2.23 | 56 |  |  | 0.01 |
|  |  |  |  |  |  |  |
| P1 | Sex | 309219.50 | 1 | 7.22 | 0.0084 | 6.50 |
|  | B chromosomes | 32771.58 | 1 | 0.76 | 0.3839 | 0.69 |
|  | Individual | 42856.15 | 103 | 17413.49 | <0.0001 | 92.80 |
|  | Error | 2.46 | 106 |  |  | 0.01 |
|  |  |  |  |  |  |  |
| P2 | Sex | 57089.69 | 1 | 1.26 | 0.2635 | 1.19 |
|  | B chromosomes | 3448.04 | 1 | 0.08 | 0.7829 | 0.07 |
|  | Individual | 45178.29 | 105 | 17598.00 | <0.0001 | 98.73 |
|  | Error | 2.57 | 108 |  |  | 0.01 |
|  |  |  |  |  |  |  |
| P3 | Sex | 125520.08 | 1 | 6.56 | 0.0137 | 12.17 |
|  | B chromosomes | 6826.32 | 1 | 0.36 | 0.5531 | 0.66 |
|  | Individual | 19130.63 | 47 | 3818.72 | <0.0001 | 87.15 |
|  | Error | 5.01 | 50 |  |  | 0.02 |
