# Supplementary material for: Canalization and developmental stability of the yellow-necked mouse (Apodemus flavicollis) mandible and cranium related to age and nematode parasitism
Source: Front Zool. 2021 Oct 24;18:55. doi: 10.1186/s12983-021-00439-4 (PMC8543932; doi:10.1186/s12983-021-00439-4)
Supplement: Supplementary file 5 — Additional file 5. Table S5 Procrustes ANOVAs of shape. % total—percentage of the total shape variation. Categories regarding parasitism: P0—non-parasitized animals, P1—animals parasitized by one nematode species, P2—animals parasitized by two nematode species, P3—animals parasitized by three to five nematode species [file 12983_2021_439_MOESM5_ESM.docx]

**Additional file 5: Table S5** Procrustes ANOVAs of shape. % total – percentage of the total shape variation. Categories regarding parasitism: P0 – non-parasitized animals, P1 – animals parasitized by one nematode species, P2 – animals parasitized by two nematode species, P3 – animals parasitized by three to five nematode species

|  | Effect | MS | df | F | P | % total |
| --- | --- | --- | --- | --- | --- | --- |
| Mandible |  |  |  |  |  |  |
| P0 | Sex | 0.0001699 | 24 | 1.04 | 0.4112 | 1.82 |
|  | B chromosomes | 0.0001814 | 24 | 1.11 | 0.3250 | 1.94 |
|  | Individual | 0.0001635 | 1032 | 5.30 | <0.0001 | 75.21 |
|  | Side | 0.0001840 | 24 | 5.96 | <0.0001 | 1.97 |
|  | Ind x Side | 0.0000309 | 1080 | 7.24 | <0.0001 | 14.86 |
|  | Error | 0.0000043 | 2208 |  |  | 4.20 |
|  |  |  |  |  |  |  |
| P1 | Sex | 0.0002495 | 24 | 1.33 | 0.1311 | 1.15 |
|  | B chromosomes | 0.0004220 | 24 | 2.25 | 0.0005 | 1.94 |
|  | Individual | 0.0001877 | 2232 | 7.00 | <0.0001 | 80.43 |
|  | Side | 0.0002481 | 24 | 9.37 | <0.0001 | 1.14 |
|  | Ind x Side | 0.0000265 | 2280 | 6.27 | <0.0001 | 11.59 |
|  | Error | 0.0000042 | 4608 |  |  | 3.74 |
|  |  |  |  |  |  |  |
| P2 | Sex | 0.0003174 | 24 | 1.77 | 0.0123 | 1.72 |
|  | B chromosomes | 0.0002645 | 24 | 1.47 | 0.0651 | 1.44 |
|  | Individual | 0.0001796 | 1992 | 7.79 | <0.0001 | 81.04 |
|  | Side | 0.0002328 | 24 | 10.09 | <0.0001 | 1.27 |
|  | Ind x Side | 0.0000231 | 2040 | 5.57 | <0.0001 | 10.66 |
|  | Error | 0.0000041 | 4128 |  |  | 3.87 |
|  |  |  |  |  |  |  |
| P3 | Sex | 0.0003515 | 24 | 1.72 | 0.0168 | 3.15 |
|  | B chromosomes | 0.0002508 | 24 | 1.23 | 0.2056 | 2.24 |
|  | Individual | 0.0002041 | 1056 | 8.10 | <0.0001 | 80.34 |
|  | Side | 0.0001187 | 24 | 4.71 | <0.0001 | 1.06 |
|  | Ind x Side | 0.0000252 | 1104 | 7.45 | <0.0001 | 10.36 |
|  | Error | 0.0000034 | 2256 |  |  | 2.84 |
| Cranium |  |  |  |  |  |  |
| P0 | Sex | 0.0000280 | 31 | 1.15 | 0.2667 | 1.72 |
|  | B chromosomes | 0.0000211 | 31 | 0.86 | 0.6832 | 1.29 |
|  | Individual | 0.0000245 | 1643 | 7.36 | <0.0001 | 79.46 |
|  | Side | 0.0000355 | 31 | 10.69 | <0.0001 | 2.18 |
|  | Ind x Side | 0.0000033 | 1705 | 5.51 | <0.0001 | 11.21 |
|  | Error | 0.0000006 | 3472 |  |  | 4.14 |
|  |  |  |  |  |  |  |
| P1 | Sex | 0.0000192 | 31 | 0.75 | 0.8440 | 0.59 |
|  | B chromosomes | 0.0000328 | 31 | 1.28 | 0.1411 | 1.01 |
|  | Individual | 0.0000257 | 3193 | 7.24 | <0.0001 | 81.31 |
|  | Side | 0.0000465 | 31 | 13.10 | <0.0001 | 1.43 |
|  | Ind x Side | 0.0000036 | 3255 | 5.47 | <0.0001 | 11.45 |
|  | Error | 0.0000006 | 6572 |  |  | 4.22 |
|  |  |  |  |  |  |  |
| P2 | Sex | 0.0000439 | 31 | 1.68 | 0.0104 | 1.31 |
|  | B chromosomes | 0.0000282 | 31 | 1.08 | 0.3484 | 0.84 |
|  | Individual | 0.0000261 | 3255 | 7.96 | <0.0001 | 81.90 |
|  | Side | 0.0000587 | 31 | 17.95 | <0.0001 | 1.76 |
|  | Ind x Side | 0.0000033 | 3317 | 5.71 | <0.0001 | 10.48 |
|  | Error | 0.0000006 | 6696 |  |  | 3.71 |
|  |  |  |  |  |  |  |
| P3 | Sex | 0.0000126 | 31 | 0.49 | 0.9921 | 0.83 |
|  | B chromosomes | 0.0000295 | 31 | 1.15 | 0.2655 | 1.96 |
|  | Individual | 0.0000257 | 1457 | 7.61 | <0.0001 | 80.20 |
|  | Side | 0.0000337 | 31 | 9.98 | <0.0001 | 2.24 |
|  | Ind x Side | 0.0000034 | 1519 | 5.91 | <0.0001 | 10.98 |
|  | Error | 0.0000006 | 3100 |  |  | 3.79 |
